# Ibero-American Endometriosis Patient Phenome: Demographics, Obstetric-Gynecologic Traits, and Symptomatology

**DOI:** 10.3389/frph.2021.667345

**Published:** 2021-06-04

**Authors:** Idhaliz Flores-Caldera, Paola M. Ramos-Echevarría, José A. Oliveras-Torres, Natasha Santos-Piñero, Estefanía D. Rivera-Mudafort, Denisse M. Soto-Soto, Brian Hernández-Colón, Luis E. Rivera-Hiraldo, Loraine Mas, Mary Rodríguez-Rabassa, Nabal J. Bracero, Edgardo Rolla, Ibero-American Endometriosis Association

**Affiliations:** ^1^Department of Basic Sciences, Ponce Health Sciences University, Ponce, Puerto Rico; ^2^Department of Ob-Gyn, Ponce Health Sciences University, Ponce, Puerto Rico; ^3^San Lucas Episcopal Medical Center, Ponce, Puerto Rico; ^4^School of Behavioral and Brain Sciences, Ponce Health Sciences University, Ponce, Puerto Rico; ^5^Department of Ob-Gyn, University of Puerto Rico, San J uan, Puerto Rico; ^6^Sociedad Argentina de Endometriosis, Buenos Aires, Argentina; ^7^Sociedad Argentina de Cirugía Laparoscópica, Buenos Aires, Argentina

**Keywords:** endometriosis, hispanic/latinx, epidemiology, symptoms, ethnicity, phenome, dysmenorrhea, dyspareunia

## Abstract

**Background:** An international collaborative study was conducted to determine the demographic and clinical profiles of Hispanic/Latinx endometriosis patients from Latin America and Spain using the Minimal Clinical Questionnaire developed by the World Endometriosis Research Foundation (WERF) Endometriosis Phenome and Biobanking Harmonization Project (EPHect).

**Methods:** This is a cross-sectional study to collect self-reported data on demographics, lifestyle, and endometriosis symptoms of Hispanic/Latinx endometriosis patients from April 2019 to February 2020. The EPHect Minimal Clinical Questionnaire (EPQ-M) was translated into Spanish. Comprehension and length of the translated survey were assessed by Spanish-speaking women. An electronic link was distributed via social media of endometriosis patient associations from 11 Latin American countries and Spain. Descriptive statistics (frequency, means and SD, percentages, and proportions) and correlations were conducted using SPSSv26.

**Results:** The questionnaire was completed by 1,378 participants from 23 countries; 94.6% had self-reported diagnosis of endometriosis. Diagnostic delay was 6.6 years. Most participants had higher education, private health insurance, and were employed. The most common symptoms were back/leg pain (85.4%) and fatigue (80.7%). The mean number of children was 1.5; 34.4% had miscarriages; the mean length of infertility was 3.7 years; 47.2% reported pregnancy complications. The most common hormone treatment was oral contraceptives (47.0%). The most common comorbidities were migraines (24.1%), polycystic ovary syndrome (PCOS) (22.2%), and irritable bowel syndrome (21.1%). Most participants (97.0%) experienced pelvic pain during menses; for 78.7%, pain was severe; 86.4% reported dyspareunia. The mean age of dysmenorrhea onset was 16.2 years (SD ± 6.1). Hormone treatments were underutilized, while impact was substantial. Pain catastrophizing scores were significantly correlated with pain intensity (*p* < 0.001).

**Conclusion:** This is the first comprehensive effort to generate a clinical–demographic profile of Hispanic/Latinx endometriosis patients. Differences in clinical presentation compared to other cohorts included higher prevalence and severity of dysmenorrhea and dyspareunia and high levels of pain catastrophizing. Though future studies are needed to dissect the impact of race and ethnicity on pain and impact, this profile is the first step to facilitate the recognition of risk factors and diagnostic features and promote improved clinical management of this patient population. The EPHect questionnaire is an efficient tool to capture data to allow comparisons across ethnicities and geographic regions and tackle disparities in endometriosis research.

## Introduction

Endometriosis is a gynecologic condition diagnosed in 6–10% of women of reproductive age, with a prevalence of 20–50% in infertile women and 71–87% of those with chronic pelvic pain (1–3). Hispanic/Latinx persons with endometriosis are underrepresented in research databases and thus unlikely to benefit from research on pathophysiology, biomarkers, and novel treatments (4). The World Endometriosis Research Foundation (WERF) Endometriosis Phenome and Biobanking Harmonization Project (EPHect) provides standardized protocols based on evidence to allow accrual of detailed surgical, clinical, and epidemiologic data from endometriosis patients to support future research. EPHect developed a clinical questionnaire to obtain self-reported demographic and clinical information to allow cross-sectional studies to better understand differences in the endometriosis phenome across populations (5–9).

A PubMed search using the keywords “endometriosis,” “ethnicity,” “Hispanic,” and “Latina” resulted in only three reports on the prevalence of endometriosis symptoms by race/ethnicity. The Nurse's Health Study II's multivariate hazard models concluded that Black [relative risk (RR) 0.6, 95% confidence interval (CI): 0.4–0.9] and Hispanic women (RR 0.6, 95% CI: 0.4–1.0) had a 40% lower rate of endometriosis diagnosis compared with White women (10). A nationwide study by Eggert et al. found rates (cases per 100,000) of endometriosis ranging from 126.2 (Asian countries) to 42.3 (Eritrea/Ethiopia/Somalia), with Latin American countries at 90.9 compared to 101.9 among Sweden-born patients (11). Both studies conclude that non-Hispanic white women have a higher likelihood of a diagnosis of endometriosis compared to other ethnic groups. However, Bougie et al. stated that there is not enough evidence to definitely conclude that the prevalence of endometriosis in Hispanic/Latinx populations is lower. In their recent systematic review and meta-analysis on the ethnic presentation of endometriosis, they identified only five studies that included patients of Hispanic/Latinx background, compared to 16 studies of White vs. Black women, and 10 studies of White vs. Asian women (12, 13). In total, these studies included only 14,951 Hispanic/Latinx patients compared to 65,332 White patients. This meta-analysis showed that endometriosis was more prevalent in Asian women (OR 1.63, 95% CI: 1.03–2.58), less prevalent in Black women (OR 0.49, 95% CI: 0.29–0.83), and less common in Hispanic women (OR 0.46, 95% CI: 0.14–1.50—not statistically significant), compared to White non-Hispanic women.

Lack of data on Hispanic/Latinx patients with endometriosis highlights the need to conduct more epidemiological investigations with diverse populations of patients to evaluate whether there are racial/ethnic differences in disease prevalence, clinical presentation, and response to treatments. More studies are needed to establish if differences in the prevalence of endometriosis and its clinical presentation among ethnic groups are due to socioeconomic factors that impact access to care, to biological/genetic correlates, or to cultural influences on healthcare seeking. To fill this knowledge gap, we aimed to obtain for the first time the clinical profile of endometriosis patients from Latin America and the Caribbean using a Spanish translation of the EPHect's Minimal Clinical Questionnaire (EPQ-M) (9). Here, we report data collected from Spanish-speaking endometriosis patients from 23 countries on demographics, lifestyle, Ob-Gyn, clinical history, pelvic pain symptoms, pain catastrophizing, impact of pain on quality of life, and utilization of pain medications and hormonal treatments.

## Materials and Methods

This study was approved by the Institutional Review Board of the Ponce Research Institute of the Ponce Health Sciences University (PHSU) (IRB Approval #1811001620).

### EPHect Survey

The EPHect's EPQ-M was designed to collect self-reported, cross-sectional data from endometriosis patients, including personal information/demographics, lifestyle and risk behaviors, obstetrical and gynecological history, fertility, medical and surgical history, pain intensity (using the numerical rating scale—NRS), pain catastrophizing, quality of life, and surgical and medical treatment. It also captures the level of pain, treatment regimen, and medication use. The EPQ-M quantifies many symptoms, including 71 quality-of-life measures and pain-related variables (McGill Pain Short Questionnaire). Confirmation of self-reported diagnosis by medical chart review was high—on average 84% overall when combining the evaluation of clinical, surgical, and pathology records (and up to 95% in some of the cohorts). These data suggest that patients with endometriosis remember with high accuracy if they have been diagnosed by a physician, especially if the diagnosis was done by surgery (14).

### Pain Catastrophizing Scale

The Pain Catastrophizing Scale (PCS) was used as a self-reported measure of catastrophic thinking (15). It evaluates three dimensions of catastrophizing: helplessness, rumination, and magnification. The items are scored from 0 (“not at all”) to 4 (“all the time”) for a total score of 52. A score higher than 30 is considered clinically relevant, and it identifies a population with a higher risk of chronicity and disability regarding pain.

### Survey Translation and Validation

A group of endometriosis experts from Latin America translated the EPQ-M survey into Spanish. A certified translator conducted a back-translation to the English version to verify its accuracy. Twenty pre-menopausal women, with and without endometriosis, assessed the comprehension, vocabulary, and length of the survey during one-on-one interviews. Anonymous responses were analyzed quantitatively and qualitatively.

### Survey Distribution

The validated Spanish EPQ-M was disseminated from April 2019 to February 2020 via a REDCap link (16) posted in social media platforms and email in collaboration with endometriosis patient support associations in Puerto Rico, Argentina, Colombia, Mexico, Panama, Costa Rica, Dominican Republic, Chile, Peru, Venezuela, and Spain. The questionnaire was self-administered, following an introductory page with instructions and IRB-specific language stating that the study was anonymous and voluntary. Data were captured electronically without identifiers.

### Data Analysis

The quality of data was evaluated and then imported to SPSSv26 (IBM Corp, Armonk, NY) for descriptive analysis. The analysis included frequencies and percentages and means and standard deviation. Pearson's correlations were calculated between PCS scores and pain intensity (dysmenorrhea, dyspareunia, and general pelvic pain) measured by the NRS. Data were subdivided into the following categories: pain and its impact, pain catastrophizing, and medical and surgical treatments. Graphical representations of data were generated using GraphPad Prism v9 (San Diego, CA). Percentages were calculated based on the number of responses per question.

## Results

### Survey Validation

Twenty participants (10 women with and 10 women without endometriosis) evaluated the survey on length, complexity, difficulty, sensitivity of questions, and usefulness of the tool for research. The mean age of evaluators was 35.4+/−7.3 years, and the majority (17/20) had at least a bachelor's degree. Most (16/20) indicated that it took them 20 min or less to evaluate the questionnaire.

#### Quantitative Data Analysis

**Figure 1:**
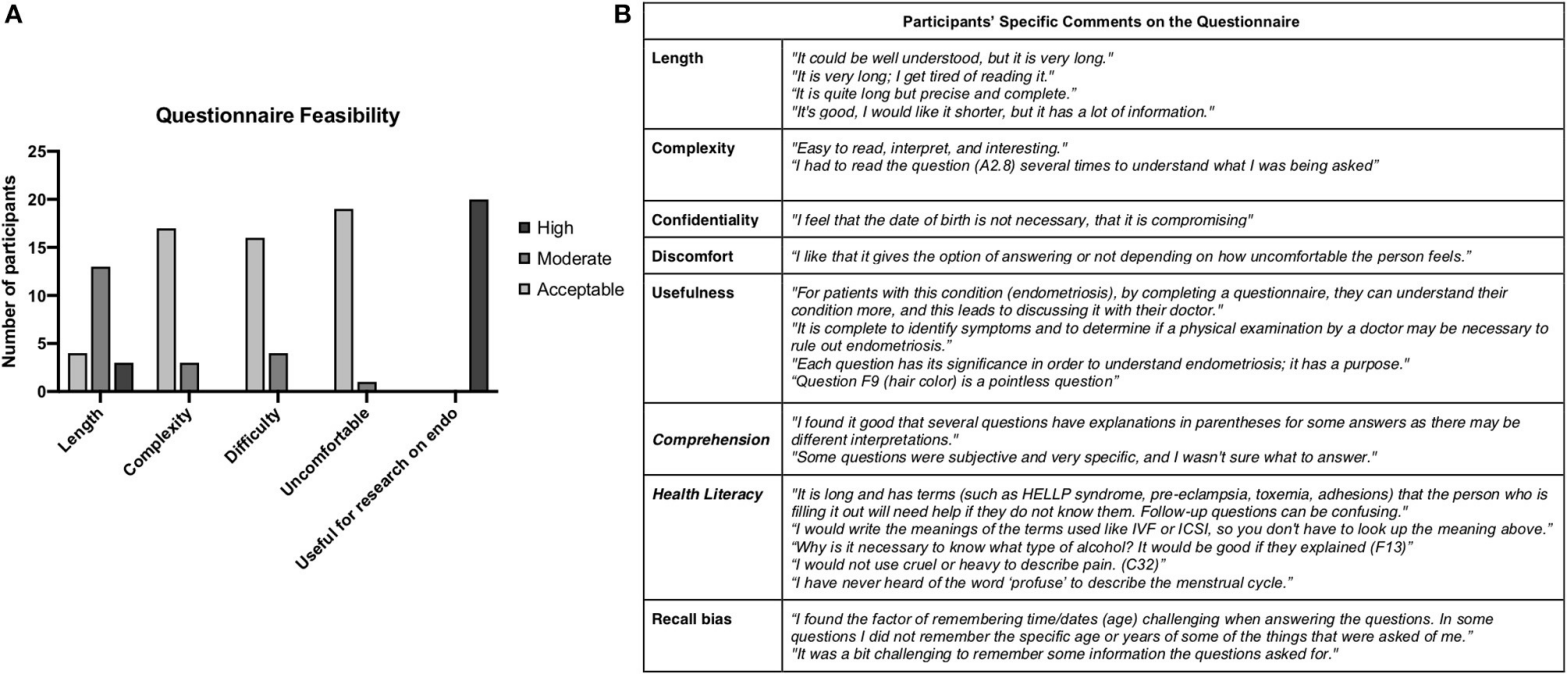
Validation of the Spanish EPhect clinical minimal questionnaire. **(A)** Quantitative assessment of feasibility. **(B)** Specific comments made by questionnaire evaluators.

The majority of the evaluators reported that the questionnaire was “moderately long” (Figure 1). Only four evaluators considered the length of the survey appropriate (all were endometriosis patients). The complexity and difficulty of the survey were well-accepted. Only one evaluator mentioned that survey included uncomfortable questions. All of the evaluators considered it useful for endometriosis-focused research.

#### Qualitative Data Analysis

Specific comments on the questionnaire are summarized in Figure 1.

### Survey Results

#### Sociodemographics

The survey linked was accessed a total of 2,352 times, and 1,378 individuals from 23 countries answered the questionnaire. Percentages were calculated based on the number of responses per question. The mean age of the participants was 33.7 (SD ± 7.2) (Table 1). The majority of participants (~80%) were employed full time or part time; 42.7% completed at least a college degree. Six of every 10 patients had private insurance.

**Table 1:** Sociodemographic characteristics of the women with endometriosis from Ibero-America.

**Characteristic**	**Mean**	**SD**
Age	33.7	± 7.2
Civil status (*n* = 1,375)	*N*	%
Married	543	39.5
Single	536	39.0
Consensual	229	16.7
Divorced	65	4.7
**Education (*****n*** **=** **1,360)**
High school	198	14.6
Associate degree	538	39.6
College	264	19.4
Post-graduate	317	23.3
**Race (*****n*** **=** **1,360)**
White	675	49.6
Black	59	4.3
Mixed race	574	42.2
Others	52	3.8
**Health insurance (*****n*** **=** **1,375)**
Private	813	59.1
Public	443	32.2
No insurance	119	8.7
**Work (*****n*** **=** **1,375)**
Employed	1,072	78.1
Full-time	271	25.3^*^
Part-time	801	74.7^*^
Unemployed	301	21.9

* *This is the percent of full-time vs. part-time within the employed participants. The real percent reflecting the work status of the full-time participants is 19.76%, and 58.3% of those who are part-time in comparison with the unemployed*.

**Figure 2:**
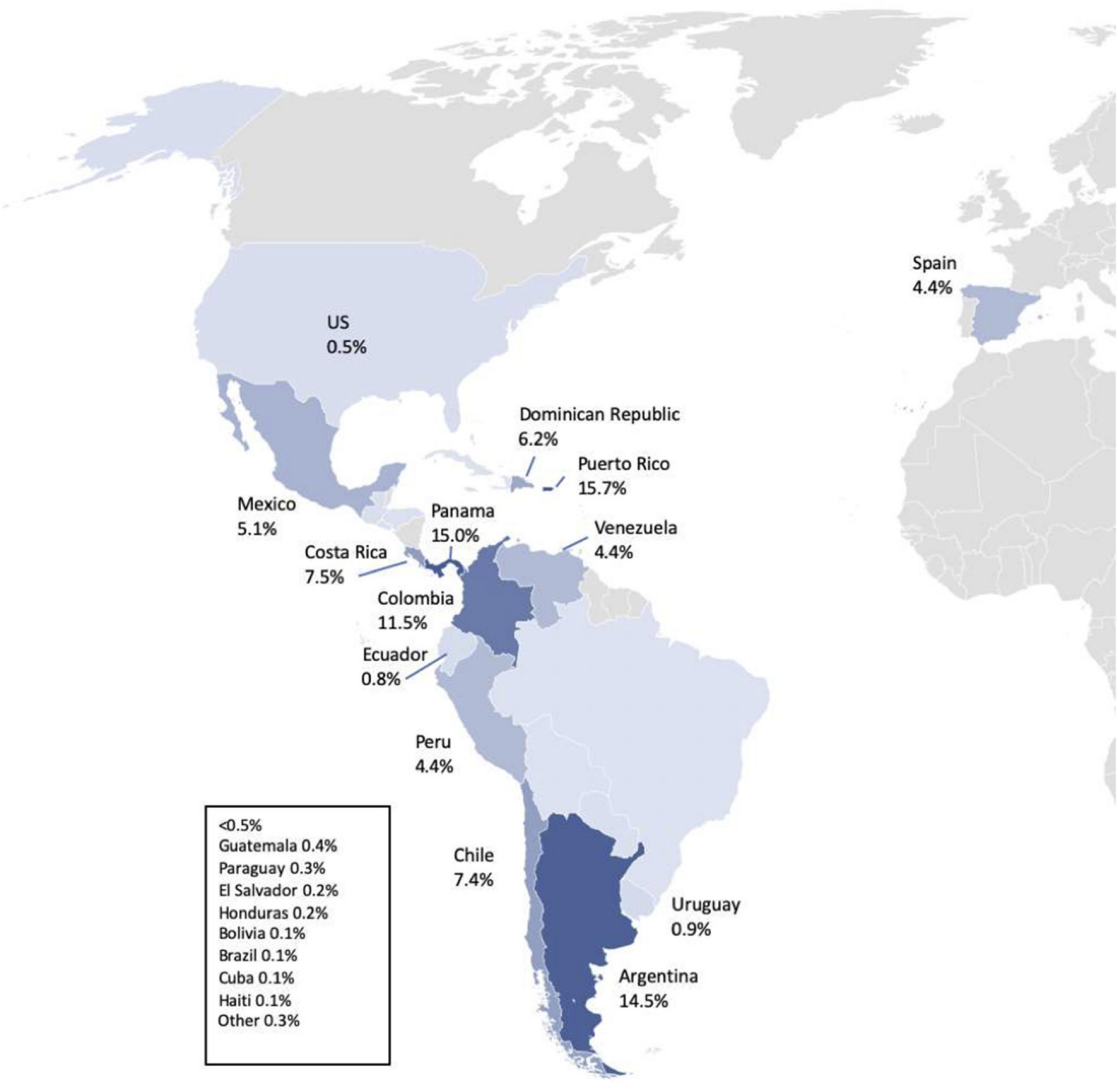
Countries of birth of study participants. Responses were received from 23 countries in South and Central America, the Caribbean, Spain, and the USA.

Almost all (98.3%, *n* = 1,323/1,346) participants self-identified as Hispanic/Latina; half (49.6%) identified as White, 42.2% as mixed race, and 4.3% as Black (Table 1). Supplementary Table 1 shows the racial distribution of participants. Most participants (15.7%, *n* = 201/1,277) were from Puerto Rico, Panama (15.0%, *n* = 191/1,277), Argentina (14.5%, *n* = 185/1,277) Colombia (11.5%, *n* = 147/1,277), and Costa Rica (7.5%, *n* = 96/1,277) (Figure 2). Patients from Chile, Dominican Republic, Mexico, Peru, Venezuela, and Spain, among other countries, also participated.

### Lifestyle Factors

#### Exercise

**Figure 3:**
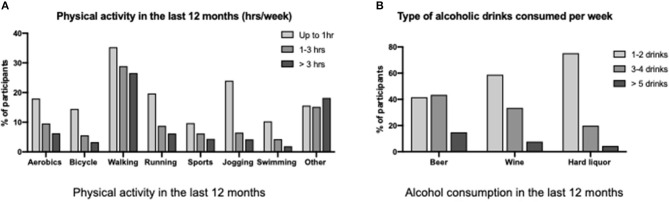
Lifestyle factors reported by participants. **(A)** Physical activity. **(B)** Alcohol consumption.

Overall, from 9.2% (walking) to 84.4% (swimming) of participants reported not exercising at all. A healthy lifestyle characterized by exercising regularly over 3 h per week was reported by 27% or less. During the previous 12 months, the top three physical activities reported were walking, jogging/running, and aerobic exercises (Figure 3A). The mean BMI was 25.3 (SD ± 5.5).

#### Risk Behaviors

Alcohol consumption was common overall (44.5%, *n* = 609/1,370) (Figure 3B). However, the risk level of alcohol consumption (over five drinks per week) was reported only by 14.8% for beer (*n* = 86/575), 7.7% for wine (*n* = 41/534), and 4.4% for hard liquor (*n* = 24/492). Regarding cigarette use, 19.7% (*n* = 271/1,375) reported to have smoked more than 100 cigarettes in their life, and only 9.4% (*n* = 129/1,371) are currently smokers.

## Gynecologic and Obstetrical History

### Menstrual Period Characteristics

The mean age at menarche of participants was 11.8 years of age (SD ± 1.61) (Table 2). Most (70.8%, *n* = 658/930) participants reported a regular cycle, with an average of 21–35 days reported by 63.2% (*n* = 565/894). Cycles of >35 days were reported by 3.7% (*n* = 33/894). On average, participants reported 6.5 days (SD ± 6.10) of menstruation. Moderate–heavy menstrual flow was reported by 84.8% (*n* = 792/934). Menorrhagia, defined as more than 7 days with a profuse menstrual flow, was reported by 11.8% (*n* = 91/768). During the prior 3 months, 30.9% (*n* = 424/1,372) reported not having menstrual periods; reasons included hormonal treatment (63.2%, *n* = 268), hysterectomy (19.1%, *n* = 81), pregnancy or breastfeeding (4.2%, *n* = 18), and menopause (4.0%, *n* = 17).

**Table 2:** Gynecologic and obstetrical traits of endometriosis patients from Ibero-America.

**Gynecologic and obstetrical traits**								
	**Mean**	**SD**			**Mean**	**Range**		
Age at menarche	11.8	±1.6	Children, N		1.5	1–5		
Age at symptoms onset	21.0	±7.8	Infertility, years		3.7	0.5–20		
Age at diagnosis	27.6	±6.7		**%**				
Diagnosis delay, years	6.6	±10.3	Miscarriages		34.4			
Age dyspareunia onset	25.1	±5.9	Pregnancy complications		47.2			
**Pregnancy history**								
**Pregnancy**	**1st**	**2nd**	**3rd**	**4th**	**5th**	**6th**	**7th**	**Total**
Mean age (years)	25.28 ± 6.0	28.47 ± 5.6	30.41 ± 5.5	31.11 ± 6.5	34.33 ± 7.0	28.33 ± 1.5	31	–
**Frequency of pregnancies**	**502**	**219**	**85**	**25**	**7**	**5**	**2**	**845**
Naturally/no fertility treatment	417	186	78	22	4	3	1	711
Artificial insemination	8	4	0	0	1	0	0	13
Clomid/clomiphene	35	21	3	1	1	1	0	62
*In-vitro* fertilization	42	8	4	2	1	1	1	59
**Pregnancy outcomes (N)**								
Babies born	310	164	54	16	4	1	0	549
Molar pregnancy	5	3	1	1	–	–	–	10
Stillbirth	5	–	2	–	1	–	–	8
Multiple pregnancy	7	3	–	–	–	–	–	10
Currently pregnant	7	3	–	–	–	–	1	11
Ectopic pregnancy	22	10	1	2	1	1	–	37
Induced abortion	46	11	11		2	1	–	71
Spontaneous abortion	101	38	23	8	2	1	–	173
**Management of ectopic pregnancy and induced/spontaneous abortion (N)**								
Oral or vaginal pills	19	10	4	1	1	–	–	35
No treatment	42	19	9	5	1	1	–	77
Dilation & curettage	73	26	12	7	2	1	–	121
**Type of delivery (N)**								
Vaginal birth	147	67	13	5	2	–	–	234
Cesarean section	182	98	41	11	3	1	–	336
No delivery	67	28	17	5	2	2	–	121
Spontaneous delivery	102	56	9	3	–	–	–	170
Induced delivery	113	45	22	8	2	–	–	190
**Infertility causes and treatments**								
**Cause (*****N*** **=** **328)**	***N*** **(%)**		Treatments^*^				**Ever** ***N*** **(%)**	**Never** ***N*** **(%)**
Endometriosis	287 (87.5)		Semen insemination from donor (*n* = 162)				5 (3.1)	157 (96.9)
Sperm quality/motility	82 (25.0)		IVF + donor eggs (*n* = 161)				15 (9.3)	146 (90.7)
Fallopian tube obstruction	80 (24.4)		IVF + ICSI (*n* = 185)				53 (28.6)	132 (71.4)
PCOS	70 (21.3)		IVF (*n* = 212)				71 (33.5)	141 (66.5)
No ovulation	49 (14.9)		Semen insemination from partner (*n* = 202)				72 (35.6)	130 (64.4)
Uterine fibroids	35 (10.7)		Progesterone (*n* = 230)				137 (59.6)	93 (40.4)
PID	29 (8.5)		Injectable fertility drugs (*n* = 243)				145 (59.7)	98 (40.3)
			Oral fertility drugs (*n* = 258)				182 (70.5)	76 (29.5)
			Timed intercourse (*n* = 274)				218 (79.6)	58 (20.4)
**Endometriosis**								
	Diagnostic method^*^			***N*** **(%)**				
	Laparoscopy/other surgery			909 (72.1)				
	Ultrasound, MRI, CT			399 (31.6)				
	Symptom-based			254 (20.1)				
	Other			110 (8.7)				

* *Participants could select more than one option. PCOS, polycystic ovary syndrome; PID, pelvic inflammatory disease; IVF, in-vitro fertilization*.

### Obstetrical History

**Figure 4:**
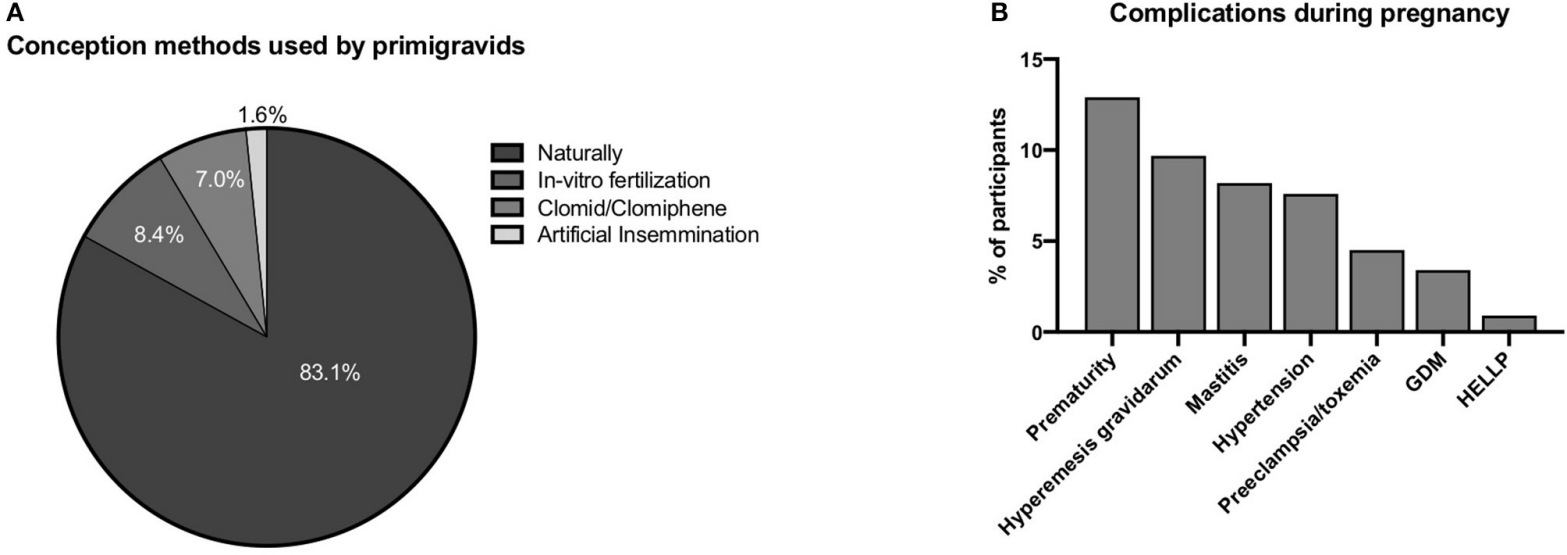
Obstetric characteristics of the study participants. **(A)** Conception methods. **(B)** Pregnancy complications.

Regardless of outcome, 37.7% (*n* = 502/1,331) of participants reported at least one pregnancy. The mean age at first pregnancy was 25.3 years (SD ± 6.00) (range: 12–41 years old) (Table 2). More than half of all pregnancies reported (59.4%, *n* = 502/845) were first-time pregnancies; of these, 83.1% (*n* = 417) were achieved naturally and 61.8% (*n* = 310) yielded live births (Figure 4A). Only eight stillbirths were reported out of a total of 845 pregnancies (0.9%). Live births were delivered most commonly by C-section (58.7%, *n* = 182/310), followed by vaginal birth (47.4%, *n* = 147/310). Of all pregnancies, 20.5% (*n* = 173/845) resulted in spontaneous abortions. Lactation length ranged from 10 to 26 months, increasing with pregnancy number.

Few molar (1.2%) or ectopic pregnancies (4.4%) were reported. Dilatation and curettage (D&C) was the treatment most often employed after spontaneous abortions (34.8%, *n* = 73/210) (Table 2). The most common obstetric complications were prematurity and hyperemesis gravidarum (Figure 4B). Rates of preeclampsia, hypertension, and gestational diabetes were 4.5, 7.6, and 3.4%, respectively.

### Infertility History

When asked if they have been trying to conceive for over 6 months, 42.9% (*n* = 488/1,137) of participants responded *yes*, with a mean time of 3.7 years (SD ± 3.2) trying (Table 2). Of those, 67.2% (*n* = 328) underwent infertility workup. Endometriosis was reported in 87.5% (*n* = 287/328) of the cases, followed by sperm quality and fallopian tube obstruction.

Methods to improve fertility were reported by 27.6% (*n* = 333/1,206) of participants (Table 2). Timed intercourse (79.6%; *n* = 218/274) and ovulation induction (70.5%; *n* = 182/258) were the most used methods. *In vitro* fertilization (IVF) was reported by 33.5% (*n* = 71/212).

### Endometriosis (Diagnosis, Family History, and Symptoms)

The majority of participants (94.6%; *n* = 1,261/1,333) reported having an endometriosis diagnosis, mostly by surgery (72.1%) (Table 2). Participants reporting a family history of endometriosis included a total of 461 (108 from mother, 113 from sister(s), 177 from a family member on the maternal side, and 123 from the paternal side, including grandmother, aunts, or cousins). A total of 1,260 participants reported a family history of chronic pelvic pain (381 from mother, 312 from a sister, 373 from a family member on the maternal side, and 194 from the paternal side).

**Figure 5:**
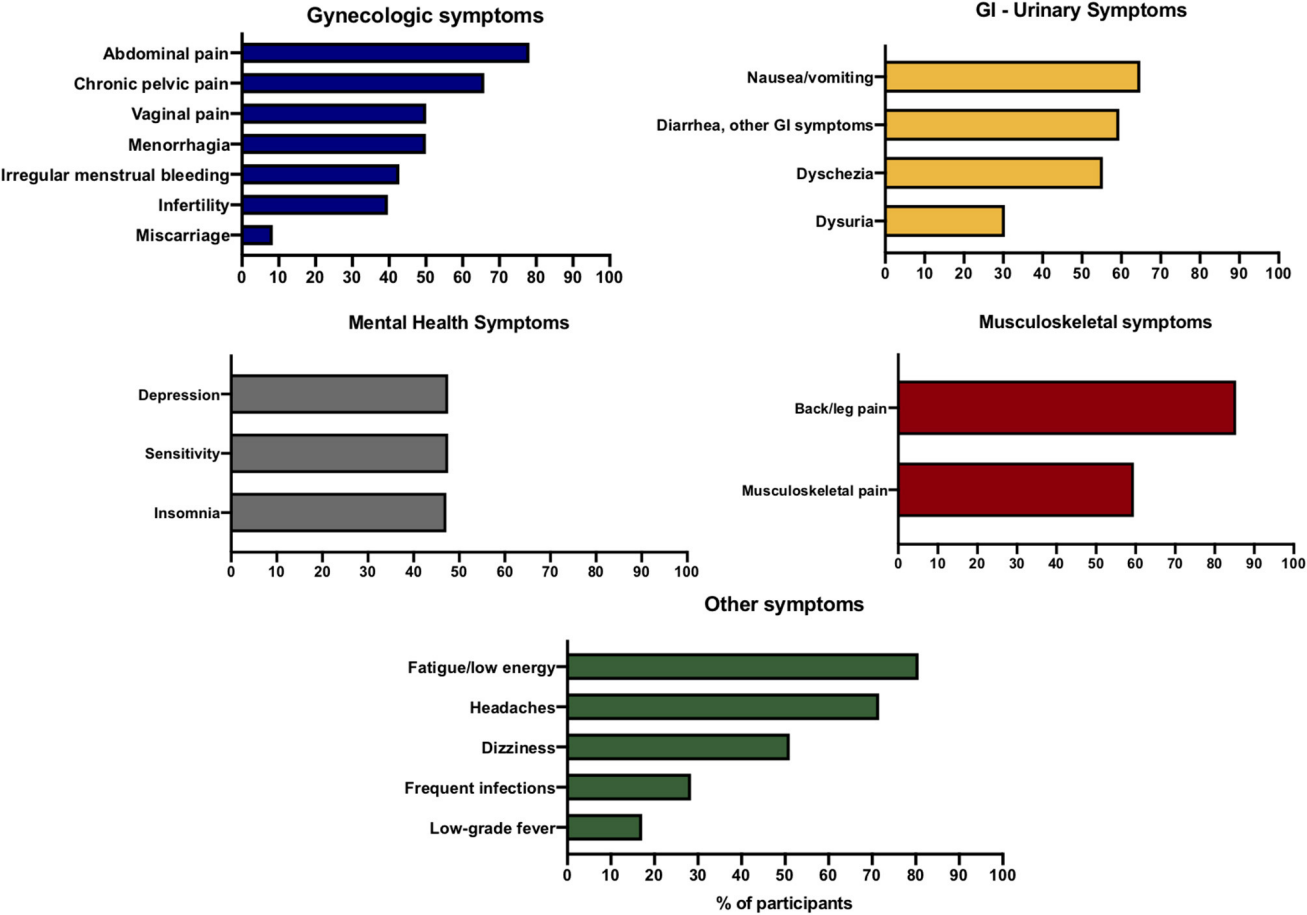
The most common symptoms reported by participants organized by system.

Most of the participants who underwent surgery for an endometriosis diagnosis were prompted by pain symptoms (89.8%, *n* = 1,132/1,261) and 25.7% (*n* = 324) for infertility or other symptoms (13.0%, *n* = 164). The mean age at pelvic pain onset was 21.9 years (SD ± 7.9 years), while the mean age at diagnosis was 27.6 years (SD ± 6.7 years). The calculated diagnostic delay for this cohort is 6.6 years on average (SD ± 10.3 years). The most common symptoms reported were back and leg pain (85.4%), fatigue and low energy (80.7%), and abdominal pain (78.2%) Participants also reported gastrointestinal symptoms, such as abdominal fullness, bloating, and swelling (82.1%), and urinary symptoms such as urgency (54.1%) (Figure 5).

### Clinical History

The most common comorbidities reported by participants were migraines, polycystic ovary syndrome (PCOS), and irritable bowel syndrome (IBS) (Table 3). Of interest, 2.3% (*n* = 31/1,322) reported a cancer diagnosis, at a mean age of 29.7 (SD ± 8.3 years). The most common self-reported malignancies were cervical (*n* = 11) and endometrial (*n* = 6) cancers. Anxiety and depression were reported by 19.5 and 17.4%, respectively.

**Table 3:** Most common comorbidities reported by endometriosis patients from Ibero-America.

**Comorbidity**	***N* (%)**	**Mean age at diagnosis (±SD)**
Migraine	332 (24.1)	21.19 ± 7.24
Polycystic ovary syndrome (PCOS)	306 (22.2)	22.88 ± 6.74
Irritable bowel syndrome (IBS)	291 (21.1)	26.03 ± 7.46
Anxiety	269 (19.5)	27.26 ± 7.98
Depression	240 (17.4)	35.06 ± 7.68
Asthma	181 (13.1)	14.38 ± 12.03
Pelvic inflammatory disease (PID)	165 (11.8)	28.00 ± 7.07
Uterine fibroids	161 (11.7)	31.05 ± 6.62
Scoliosis	154 (11.2)	19.18 ± 8.63
Thyroid	142 (10.3)	28.27 ± 8.27
Fibromyalgia	82 (6.0)	32.08 ± 8.04
Interstitial cystitis	58 (4.2)	26.76 ± 8.37
Other spinal disease	54 (3.9)	28.75 ± 10.60
Chronic fatigue syndrome	51 (3.7)	32.16 ± 7.90
Heart disease	48 (3.5)	24.93 ± 11.60
Hashimoto's disease	40 (2.9)	32.43 ± 8.82
Diabetes	38 (2.8)	34.89 ± 7.54
Rheumatoid arthritis	32 (2.3)	30.16 ± 8.73
Mitral valve prolapse	30 (2.2)	22.38 ± 8.64
Eczema	23 (1.7)	21.55 ± 8.72
Systemic lupus erythematosus	14 (1.0)	27.21 ± 6.10
Ulcerative colitis	13 (0.9)	26.00 ± 6.51
Deafness	11 (0.8)	26.40 ± 17.83
Sjogren's syndrome	8 (0.6)	29.13 ± 11.13
Multiple sclerosis	3 (0.2)	41.00 ± 2.83
Crohn's disease	2 (0.1)	38.00 ± 2.83
Glandular fever	2 (0.1)	26.50 ± 7.78
Graves' disease	1 (0.1)	30.00

### Non-Menstrual Pelvic Pain

**Figure 6:**
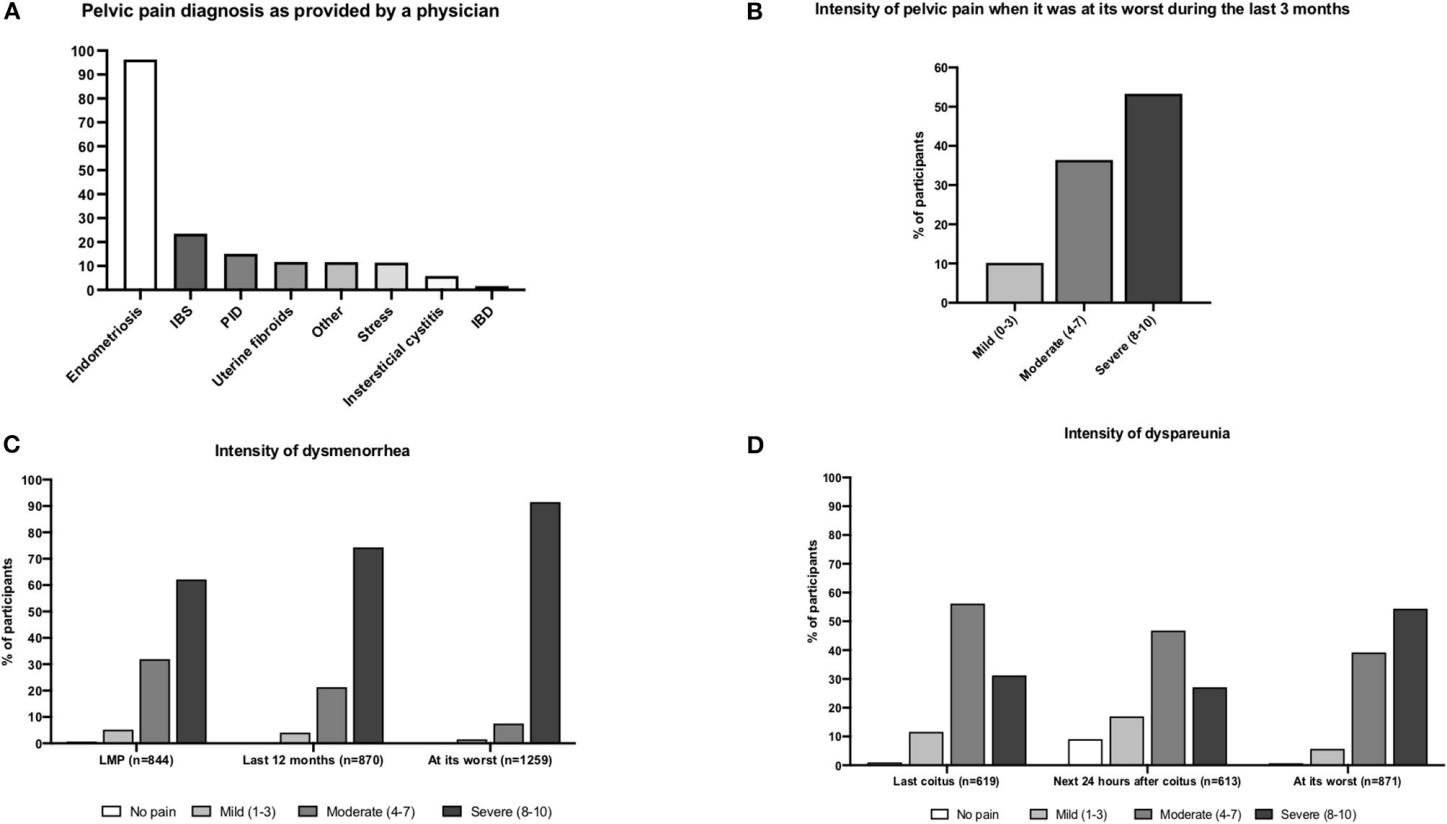
Diagnosis and intensity of pelvic pain. **(A)** Pelvic pain diagnosis. **(B)** Intensity level of pelvic pain (last 3 months). **(C)** Intensity of level of dysmenorrhea. **(D)** Intensity level of dyspareunia.

The majority (94.6%; *n* = 1,261/1,333) of participants had a self-reported diagnosis of endometriosis. Their mean age at pelvic pain onset was 21.9 (SD ± 7.9 years). The majority (84.7%, *n* = 993/1,173) reported experiencing generalized pelvic pain *at some point during their life*; 63.6% (*n* = 604/949) reported experiencing pelvic pain *during the past month*. Most participants (87.9%, *n* = 809/920) stated that the etiology of the pelvic pain was diagnosed by a physician. The most common diagnoses for pain were endometriosis (96.3%, *n* = 779), IBS (23.5%, *n* = 190), and PID (15.1%, *n* = 122) (Figure 6A). Participants often reported these conditions concomitantly.

Many participants (78.0%) reported feeling pelvic pain *within the last 3 months* (*n* = 740/949). This pain was mostly aggravated by stress (58.1%, *n* = 430), voiding or having a full bladder (58.0%, *n* = 429), and constipation (55.3%, *n* = 409). It was mostly relieved by pain medications (79.7%, *n* = 590) or laying down (72.2%, *n* = 534). Pain intensity *during the last 3 months* was reported as severe by 53.3% (*n* = 391/734) of participants (Figure 6B).

### Pelvic Pain During Menses (Dysmenorrhea)

The majority of participants (97.0%, *n* = 1,320/1,361) reported experiencing “pelvic pain *during menses* at some point during their life.” The pain was severe for 78.7% (*n* = 1,039) and moderate for 16.1% (*n* = 213). The mean age of dysmenorrhea onset was 16.19 ± 6.09 years. Dysmenorrhea was experienced during the last 12 months by 59.7% (*n* = 522/874) of participants. Severe pain intensity during the *last menstrual period* was reported by 62.2% (*n* = 525/844) and moderate by 31.9% (*n* = 269/844). *During the past 12 months*, 74.3% (*n* = 646/870) described their pain intensity as severe and 21.3% (*n* = 185/870) as moderate. When dysmenorrhea *was at its worst*, 91.5% (*n* = 1,152/1,259) reported severe pain, while only 7.5% (*n* = 94/1,259) reported moderate pain (Figure 6C). The mean pain intensity for dysmenorrhea ranged from 7.7 to 9.2 (Table 4).

**Table 4:** Summary of pain intensity measured by NRS.

**Intensity of…**	***N***	**Mean score**	**SD**
Pelvic pain during the last 3 months	734	7.43	± 2.14
Dysmenorrhea during last menstrual period	844	7.66	± 2.19
Dysmenorrhea during last 12 months	870	8.22	± 2.09
Dysmenorrhea at its worst	1,259	9.19	± 1.36
Dyspareunia during last coitus	619	6.19	± 2.24
Dyspareunia during the next 24 h after coitus	613	5.30	± 2.84
Dyspareunia at its worst	871	7.32	± 2.21

### Pelvic Pain During Sexual Intercourse (Dyspareunia)

Most of the participants (86.4%, *n* = 907/1,050) had experienced dyspareunia throughout their lives, with a mean age of onset of 25.1 ± 5.85 years of age. Most (77.8%, *n* = 619/796) reported experiencing dyspareunia during their *last sexual encounter*. The pain was mostly felt during coitus (40.2%, *n* = 249), within the first 24 h afterwards (18.6%, *n* = 115), or both (41.2%, *n* = 255). Up to 75.9% (*n* = 44/58) admitted having avoided coitus due to the dyspareunia. The pain was generally experienced during the days following menses (34.9%, *n* = 97) and was most commonly localized in the pelvis or abdomen (43.9%, *n* = 457) and the deep vaginal canal (39.2%, *n* = 408).

Considering dyspareunia *during the last year*, most (72.4%, *n* = 440/608) of the participants interrupted coitus due to pain and 82.5% (*n* = 504/611) reported having avoided coitus overall. In general, 54.4% (*n* = 474/871) of participants classified dyspareunia as severe when it was at its worst.

Pain intensity (Figure 6D) during the *last sexual intercourse* (*n* = 619) was severe for 31.2% (*n* = 193) and moderate for 56.2% (*n* = 348). During the *24 h after intercourse* (*n* = 613), pain intensity was severe for 27.1% (*n* = 166/613) and moderate for 46.8% (*n* = 287). Dyspareunia *at its worst* (*n* = 871) was severe for 54.4% (*n* = 474) and moderate for 39.2% (*n* = 341). The mean pain intensity for dyspareunia ranged from 5.3 to 7.3 (Table 4).

**Figure 7:**
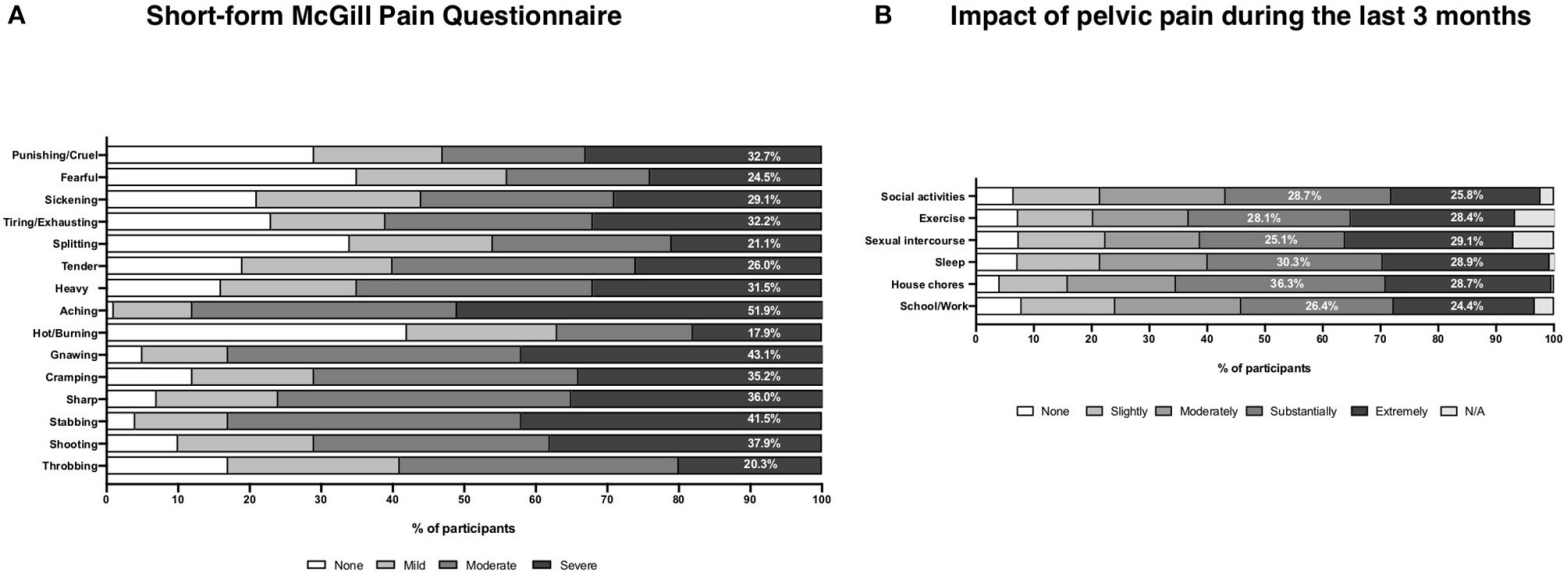
Pain characteristics and impact of pelvic pain on lifestyle. **(A)** Pain characteristics by the McGill Pain Questionnaire Short Form results. **(B)** The impact of pelvic pain during the last 3 months.

Most participants (92.7%, *n* = 856/923) reported taking medication for pelvic pain control. Approximately half of them used over-the-counter (OTC) medications (52.5%, *n* = 449/856), while 39.0% (*n* = 334/856) used prescribed medications. Few participants reported hormone use (8.3%, *n* = 73/856) as a therapeutic option for the pain, of which only 49.3% (*n* = 36/73) reported pain relief. A total of 735 participants completed the SF-MPQ. The mean score for this questionnaire was 22.30 (SD ± 11.59). Pelvic discomfort was mostly described as severely painful (46.9%, *n* = 345), persistent (38.0%, *n* = 279), and stinging (37.7%, *n* = 277) (Figure 7A).

### Impact of Pain in Daily Activities

Participants reported the pain being substantially (25.1–36.3%) or extremely (24.4–29.1%) interfering in daily activities, such as house chores, sleep, exercise, social activities, sexual intercourse, and school/work (Figure 7B).

### Pain Catastrophizing Scale (PCS)

Out of all the participants, 1,275 completed all the questions of the PCS. Of those, 45.8% (*n* = 585) obtained a total score of 30 or higher, and 24.5% (*n* = 313) had a score between 20 and 29. The mean score for this scale in our population was 27.9 (SD ± 13.66*)* (Table 5), which is higher than what has been established as the 50th percentile in previous studies on endometriosis patients (17). The median in our cohort was 28.0, comparable to what has been reported in another cohort (18). Within each subscale of the PCS, there was one expression that was scored significantly higher than the others (*p* < 0.001): helplessness subscale: “I worry all the time about whether the pain will end,” mean score of 2.45 (SD ± 1.26); magnification subscale: “I become afraid that the pain will get worse,” mean score of 2.63 (SD ± 1.32); rumination subscale: “I anxiously want the pain to go away,” mean score of 3.05 (SD ± 1.23). Additionally, there was a positive correlation between the PCS score and pain symptomatology: pain intensity in dysmenorrhea at the last menstrual period (slope = 2.67, Pearson's *r* = 0.44, *p* < 0.001), dysmenorrhea at its worst (slope = 2.16, Pearson's *r* = 0.15, *p* < 0.001), dyspareunia at its worst (slope = 1.35, Pearson's *r* = 0.23, *p* < 0.001), and general pelvic pain at its worst in the last 3 months (slope = 1.54, Pearson's *r* = 0.30, *p* < 0.001).

**Table 5:** Pain catastrophizing scale and subscales results.

	**Mean ± SD**	**Range**	50th percentile score^*^	75th percentile score^*^	Third quartile *n* (%)^**^	Fourth quartile *n* (%)^**^
**Pain catastrophizing scale results (*****n*** **=** **1,275)**
Rumination	8.94 ± 4.60	0–16	9	13	289 (22.6%)	492 (38.6%)
Magnification	6.44 ± 3.54	0–12	7	9	222 (17.4%)	847 (66.4%)
Helplessness	12.50 ± 6.68	0–24	12	18	295 (23.1%)	635 (49.8%)
Overall	27.86 ± 13.66	0–52	28	38	313 (24.5%)	585 (45.8%)

* *The score was obtained by performing an interquartile analysis within the results of our cohort*.

** *Statistics were calculated for the scores established by Sullivan et al. for the 50th and 75th percentile in a population with chronic pain. The 50th percentile was a score of 8 for rumination, 3 for magnification, 8 for helplessness, and 20 for the overall score. The 75th percentile was a score of 11 for rumination, 5 for magnification, 13 for helplessness, and 30 for the overall score*.

### Surgical Procedures

At least one abdomino-pelvic diagnostic laparoscopic procedure was reported by 61.6% (*n* = 849/1,378) of participants, while 14.7% (*n* = 202) reported having undergone at least two. The mean number of laparoscopies was 1.61 (± 1.01), with a range from 1 to 8. The mean age of laparoscopy was 27.54 ± 6.56 years, and the mean age at hysterectomy was 36.97 ± 6.29 years.

### Pain Medications

During the *worst pain episodes* of dysmenorrhea, the most commonly used medications were OTC (63.0%, *n* = 831/1,320) and prescribed analgesics (43.5%, *n* = 574/1,320). Fewer participants (21.6%, *n* = 285/1,320) reported having used hormone-based medications for the pain, which were beneficial for less than half of them (46.7%, *n* = 133). Despite the use of analgesics, 42.3% (*n* = 358/846) of those experiencing dysmenorrhea during their last menses reported that the pain had affected their daily activities.

**Figure 8:**
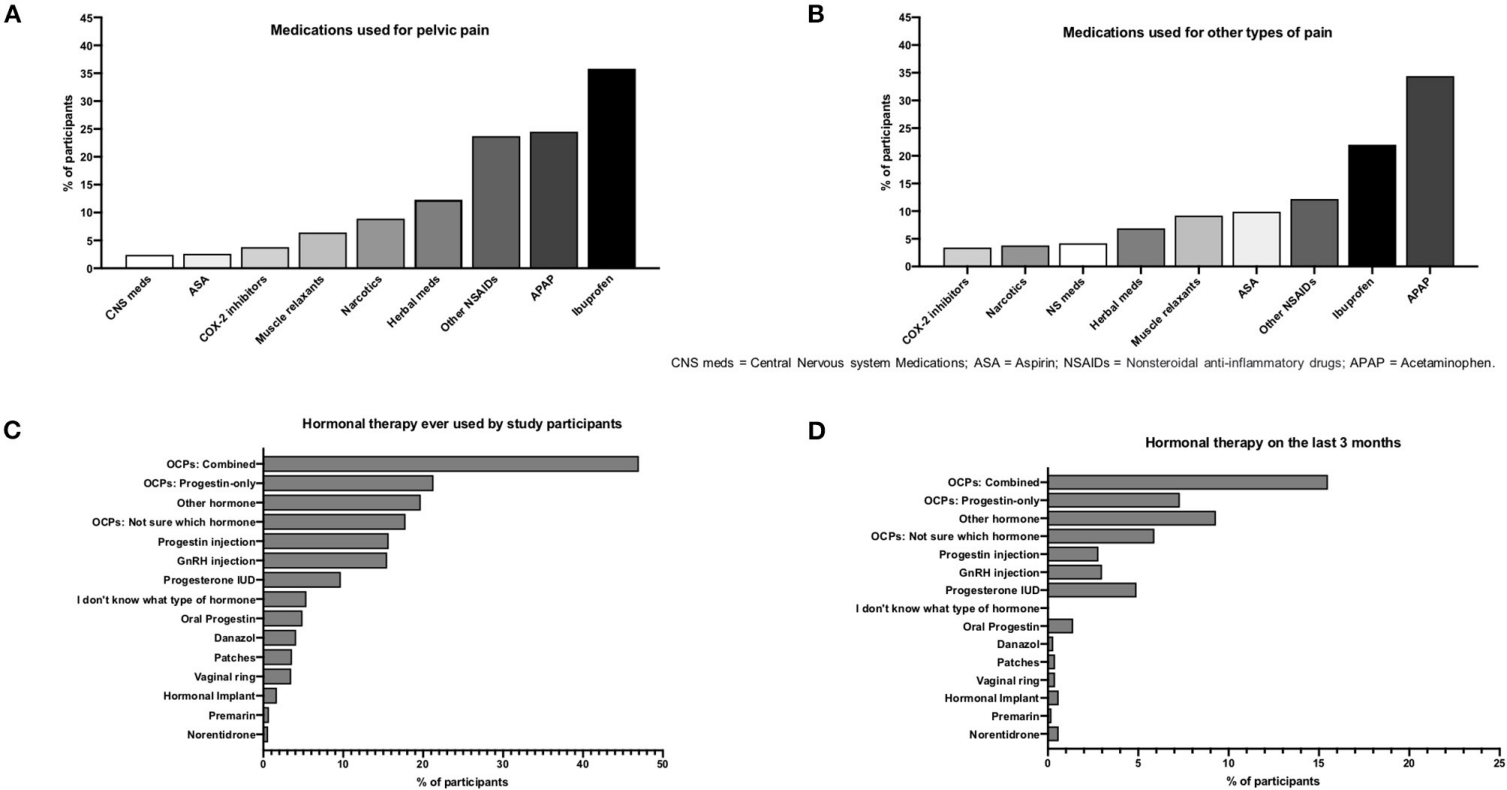
Pain medications and hormonal therapies. **(A)** Medications used for pelvic pain. **(B)** Medications used for other types of pain. **(C)** Hormonal therapies ever used by participants. **(D)** Hormonal therapies during last 3 months.

The most common medications used for pelvic pain were ibuprofen (35.8%, *n* = 493), acetaminophen (24.5%, *n* = 337), and other NSAIDs (23.7%, *n* = 327) (Figure 8A). Similarly, these agents were reported as the most common medications used for other types of pain (*n* = 1,461) (acetaminophen: 34.4%, *n* = 474; ibuprofen: 22.0%, *n* = 303; other NSAIDs: 12.2%, *n* = 168) (Figure 8B).

### Hormonal Medications

Oral contraceptive pills (OCPs) were the hormonal therapy used the most, reported by 47.0% (*n* = 647) of participants (Figure 8C) and by 15.5% (*n* = 213) during the last 3 months (Figure 8D). OCPs were used for the longest time (56 ± 65.4 months on average). The main reason for hormone therapy was treatment for pelvic pain (55.4%; *n* = 764) followed by contraception (28.0%; *n* = 386). Only 40.7% (*n* = 309/760) reported some type of improvement of pain after OCP treatment. Half of the participants (50.4%; *n* = 225/446) changed treatment methods due to failed symptomatic relief of pelvic pain.

## Discussion

This study was conducted to determine, for the first time, the demographic and clinical profile of endometriosis patients from Ibero-America using the Spanish translation of the EPhect's Minimal Clinical Questionnaire (EPQ-M) (19). Also, we demonstrate that the EPHect questionnaire is an efficient tool for standardized self-reported data collection to allow comparisons of the endometriosis phenome across ethnicities and geographic regions (5). The scarcity of investigations on the prevalence, disease presentation, level of impact, access to health care, and treatments in endometriosis patients who identify as Hispanic/Latinx adds to the current belief that this disease is less prevalent than in non-Hispanic White or Asian women (20). Collecting comprehensive data from patient populations not well-represented in databases is also critical to understand if there are differences in the clinical presentation of endometriosis based on race, genetics, and environmental or cultural factors and ultimately could help solve disparities in their representation in endometriosis research (4).

The translated EPQ-M survey was well-understood and accepted by Spanish-speaking women. Most evaluators considered it to be too long but a useful tool to conduct research on endometriosis across populations. Cross-sectional data was then collected from 1,378 Hispanic/Latinx endometriosis patients, recruited through collaborations with patient associations from South America, Central America, the Caribbean, and Spain, which represents the largest patient cohort from this ethnic background. Though the study link was disseminated through well-organized patient associations, the sample size could be considered small. Since prevalence data of endometriosis in Latin American and Caribbean populations are lacking (21, 22), it is difficult to estimate the generalizability of our study findings. Based on self-reported race, our cohort was 49.6% White, 4.5% Black, and 42.1% mixed race, vs. the estimated racial distribution of Latin American and Caribbean populations of 11% indigenous, 20% Afro descendants, and 40% White, in average, and these proportions vary from country to country (23). On closer look, the distribution of races per country in our study in general is representative of the respective racial proportions, except for Colombia, Mexico, and Venezuela. For example, Spain, Uruguay, and Argentina had the highest proportions of White participants, while countries with high Amerindian heritage such as Ecuador, Perú, and Mexico had the highest proportions of mixed race (Supplemental Table 1). Also, as expected, Black women had the highest participation in Dominican Republic, Panamá, and Puerto Rico, three countries with rich African heritage. Notably, the study had appropriate representation of non-White women (46.6% overall, and either equal to or higher than expected in most countries), who are not often well-represented in research cohorts. Efforts to recruit more diverse patients are ongoing, and future studies to assess the impact of race and ethnicity on endometriosis symptomatology are warranted.

Our study population had a relatively high economic status, the majority being employed and many having a higher degree of education. The level of education of our cohort is within that reported in other studies, ranging from 45 to 85% of patients reporting a higher than high school education (10, 24–26). It has previously been noted that there is an increased prevalence of endometriosis among well-educated women of higher socio-economic status, which may be due to better access to care or health awareness or possibly other lifestyle factors or habits linked to endometriosis risk (27).

Health risk behaviors were uncommon in this patient cohort; <15% reported high levels of alcohol consumption and <10% were smokers. Correlations between current alcohol intake and smoking and endometriosis risk (in infertile women) have been reported (10). A recent meta-analysis, however, did not find any association between smoking and endometriosis risk (28). Overall, our population did not exercise regularly: only around 30% reported exercising weekly. Moderate-to-high intensity physical activity has been associated with reduced endometriosis risk (29), though randomized studies to establish if an exercise intervention is effective against endometriosis are lacking. Thus, while this cohort did not report high rates of risk behaviors, they also had low rates of protective behaviors against endometriosis development.

The mean age of the participants falls within the age groups with the highest incidence of endometriosis (24–36 years of age) (10). Our cohort reported a lower age at menarche compared to others (11.8 vs. 12.8–12.9 years of age) (28, 30, 31). Menorrhagia prevalence (11.2%) falls within what has been previously reported (2.3–13.2%) (32, 33). We identified known risk factors for endometriosis in this population, including early age at menarche, long and heavy menstrual cycles, and nulliparity (62.3%). The average number of children was 1.5, which is similar to another study (1.7 ± 1.1) (34). The prevalence of spontaneous abortions (20.5%) was within that reported by other endometriosis patient cohorts (14.3–20.8) (24, 34) and that of women without endometriosis (34). The rate of C-sections in our cohort (58.7%) was higher than the US national average (31.9% in 2018) (35) and other patient cohorts of (37.3%) (36, 37). Stillbirth rate (0.9%) was lower than that reported in other endometriosis cohorts (1.9%), while ectopic pregnancy rate was higher (4.4%) compared to another study (1.8%) (34).

Endometriosis has been associated with adverse maternal, fetal, and neonatal outcomes (34, 36, 38). Yet, in our cohort, we observed preeclampsia rates (4.5%) that are within the global rate of 2–8% (39) and lower than the rate reported by other cohorts of endometriosis patients (7.9 and 9.5%) (34, 36). The rate of gestational diabetes (3.4%) was also lower when compared to the average rate in the USA (6%) (39) and to other studies of endometriosis patients (4.3%) (34). Notably, a substantial proportion of our cohort had sought infertility treatment (67.2% of those ever trying to get pregnant), which is higher than that reported in other cohorts (33.7 and 52.8%) (28, 34). We calculated 3.7 years with infertility in average. Endometriosis was reported as the primary cause of infertility by 87.5%, much higher than that in other cohorts (52.8%) (31, 34). The utilization rate of *in vitro* fertilization (IVF) was 8.4%, similar to other cohorts (7.7%) (36, 37).

In average, the diagnosis of endometriosis was delayed 7 years, in accord with previous estimates (40). The primary reason for diagnostic surgery was for pelvic pain (>90%), higher than other cohorts (63 and 21%) (28, 33). Infertility as the reason for surgery (26%) was within what has been reported in other studies (4–55%) (28, 33). Overall, this study did not identify any major differences in the patient profile compared to other patient cohorts. The most common symptoms reported were back and leg pain, bloating, and fatigue, which were higher than more characteristic symptoms of endometriosis such as pelvic pain (30, 41). GI and urinary symptoms were also very commonly reported as in other cohorts (42).

The most common comorbidities self-reported in our cohort were migraines, PCOS, and IBS. Although endometriosis patients are more likely to suffer migraine headaches (43), the prevalence of migraines in our cohort (24.1%) is close to the upper range for the lifetime prevalence of migraine in women (20.0%; 95% CI: 16.6–23.8%) (44) and lower than in other patient cohorts (35.2 and 38.3%) (45, 46). The PCOS rate (22.2%) was higher than the worldwide incidence (3–10%) (47) and higher than in other cohorts (5%) (48). The IBS prevalence was higher in our study (21%) than what is reported in the US population (14.1%), but similar to other patient cohorts (22.4 and 24.0%) (49, 50). The prevalence of uterine leiomyomas was 12%, which is within what has been reported in other patient cohorts (5–21%) (24, 28, 33, 48) and lower than in the US population (35%) (51). The self-reported prevalence of cancer was relatively high (2.3%) for a young population (mean 33.7 years old); this rate is higher than what has been reported in another cohort (ovarian−0.2%, breast−0.4%, and melanoma−0.7%) (52). Rates of anxiety (19.5%) and depression (17.4%) were significantly lower than those reported in another cohort (45% anxiety and 30% depression) (53).

The majority of patients in this cohort reported feeling generalized pelvic pain early in their lives (in their 20's); endometriosis was the most common cause of this pain, followed by IBS and PID. Dysmenorrhea was reported by almost all participants (97%), and 79% considered the pain severe. This prevalence is higher than that in other studies (69%) (30, 31). Our patient cohort has been extremely affected by pelvic pain as 9 in 10 patients described their pain at its worst as being between intensity 8 and 10 on the NRS. The prevalence of dyspareunia (86.4%) was higher than that in other patient cohorts (38 and 45%) (30, 31), and 8 out of 10 reported severe symptomatology during their last sexual encounter. Many participants (49–65%) reported a substantial-to-extreme interference of daily activities by their pain, adding to the strong evidence that endometriosis impacts all aspects of the life course of those affected (54).

While it identified that there is access to surgical expertise across the region (55), our study was not designed to detect whether this access was determined by socioeconomic status or other variables. In fact, the EPQ-M survey does not capture information on income, urban/rural residence, or other known proxies of economic status when in fact these factors have been shown to influence access to standard of care in endometriosis. A study conducted in Puerto Rico showed that patients of endometriosis who had public health insurance were less likely to receive gynecologic consultation and more likely to seek emergency room services (56). Follow-up studies on factors that affect access to care in each country are needed to better understand and be able to solve any disparities.

This cohort used hormonal treatments (mainly OCP) predominantly for pain management, yet their use was reported by a relatively low number of participants (only half ever used them and 16% during the last 3 months). This indicates that there are opportunities to improve access to hormonal medications that have been shown to provide substantial endometriosis-related pain relief. It will be important to investigate whether cost limits access to hormonal medications or if cultural factors, such as taboos, religion, and stigma, can play a role in the low use of hormones that are also prescribed for contraception. Another important consideration is the high rate of treatment failure to hormonal drugs observed in this study and others that may impact treatment adherence (30).

The average age at diagnosis of our cohort was 22 years old. Patients' age has a profound impact in the management of endometriosis since modern trends avoid surgery and the use of advanced hormonal treatments during early reproductive years. Experts are leaning more toward a presumptive diagnosis of endometriosis trying to avoid multiple laparoscopies and toward combined oral contraceptives that are better tolerated and can be prescribed for a longer, indefinite time. In contrast, most advanced hormonal therapies such as GnRH analogs can only be used for 6–24 months. Furthermore, this young age for self-awareness of endometriosis may reflect a success story regarding patients' self-education and potential providers' detection in timely fashion leading to improved control of the condition.

One of the key symptoms that patients with endometriosis present is pelvic pain, which can become chronic and is often refractory to treatments. This debilitating symptom is cause for substantial detriments to the physical functioning, emotional wellbeing, and quality of life (57–59). We showed here high rates of all pain manifestations surveyed (dysmenorrhea, dyspareunia, and non-menstrual pain) compared to published data. Notably, negative coping responses to painful experience such as pain catastrophizing were common in this patient population. We also observed a positive correlation between reported pain levels and catastrophizing scores, as shown before (18, 60). This maladaptive mechanism has been associated with pain persistence and correlated with poorer quality of life and depression, and it may impact the clinical management of pelvic pain (15, 17, 18). Based on the Pain Catastrophizing Scale score, 46% of our cohort has a high risk of developing persistence of their pain and disability (PSC > 30) (18). This supports the importance of applying a psychosomatic approach for clinical management of chronic pain in endometriosis patients (61). Follow-up studies to assess the impact of race, ethnicity, and socioeconomic factors on the pain catastrophizing scores in this patient population are warranted.

This study may not be representative of all women with endometriosis in this population for various reasons. There may be a bias of recruitment through patient organizations as it is possible that members are more symptomatic. Collection of self-reported data has been previously shown to have relatively high concordance with surgical diagnosis (14). Our study population was highly educated, which may be explained by the fact that the study was conducted using an electronic link requiring of internet access. This finding is in accord to studies showing increased prevalence of endometriosis among well-educated women of higher socio-economic status (27). While, overall, the race distribution in each of the countries included was in accord with each country race proportions, there was a lower-than-expected participation of Black patients. Thus, it could be argued that these results are not representative of all endometriosis patients in this world region. Follow-up studies must be conducted to include a wider racial, ethnic, and socioeconomic representation of persons with endometriosis in these countries, to assess the impact of these factors on pain perception and impact. Our study highlights the need to establish a centralized repository of data collected longitudinally using the EPHect tools that are publicly available for future research, possibly with funding from local institutes of health, patient associations, gynecologic practices, and the WERF.

In conclusion, this study uncovered for the first time the phenome of endometriosis patients of Hispanic/Latinx background, showing substantial severity of symptoms, high pain catastrophizing scores, and overall negative impact on quality of life. As EPHect tools become more widely used, future studies must take into account confounding variables such as socioeconomic status, culture, race, and health systems, as these are known to impact access to care and thus could directly affect the clinical diagnosis of endometriosis. Such studies should also address cultural and religious factors regarding menstruation, contraception, and women's health issues, including normalization of symptoms and paternalistic nature of patient–doctor interactions (62). Importantly, forthcoming studies must recognize that the Hispanic/Latinx population is heterogeneous genetically and culturally and that there are differences in health insurance systems, availability/access to medications or surgical procedures, referral trends for sub-specialists, exposures, diet, and other environmental factors/stressors. Together, all these factors influence clinical care, disease presentation, and outcomes; thus, more research is needed to disentangle their contributions and to provide the best medical care possible to all patients, irrespective of race/ethnicity.

## Data Availability Statement

The raw data supporting the conclusions of this article will be made available by the authors by request.

## Ethics Statement

The studies involving human participants were reviewed and approved by Ponce Research Institute Institutional Review Board (IRB). Written informed consent for participation was not required for this study in accordance with the national legislation and the institutional requirements.

## Author Contributions

IF-C made substantial contributions in the conception and design of the study, data acquisition, analysis and interpretation of data, drafting the article, critically revising the manuscript for important intellectual content, and giving the final approval. PR-E made substantial contributions in the analysis and interpretation of data, drafting and revising the article, and giving the final approval. JO-T, NS-P, ER-M, BH-C, LR-H, and LM made substantial contributions in the analysis and interpretation of data, revising the article, and giving the final approval. DS-S made substantial contributions in the analysis and interpretation of data, revising the article, and giving the final approval of the version to be published. MR-R made substantial contributions to the conception and design of the survey validation study, revising the manuscript critically for important intellectual content, and giving final approval. NB and ER made substantial contributions in the analysis and interpretation of data, revising the manuscript critically for important intellectual content, and giving the final approval. The Ibero-American Endometriosis Association^*^ made substantial contributions to the acquisition of data, revision of the article critically for important content, and the final approval. All authors contributed to the article and approved the submitted version.

## Conflict of Interest

The authors declare that the research was conducted in the absence of any commercial or financial relationships that could be construed as a potential conflict of interest.
