# Supplementary material for: Ibero-American Endometriosis Patient Phenome: Demographics, Obstetric-Gynecologic Traits, and Symptomatology
Source: Front Reprod Health. 2021 Jun 4;3:667345. doi: 10.3389/frph.2021.667345 (PMC9580711; doi:10.3389/frph.2021.667345)
Supplement: Supplementary file 1 [file Data_Sheet_1.PDF]

Supplemental Table 1: Racial distribution of endometriosis patients by country				
Country	**Race n (%)****			
	White	Mixed	Black	Other
**Spain** (n=65)	64 (98.5)	-	1 (1.5)	-
**Uruguay** (n=16)	15 (93.8)	-	1 (6.3)	-
**Argentina** (n=212)	175 (82.5)	35 (16.5)	-	2 (0.9)
**Chile** (n=101)	79 (78.2)	16 (15.8)	1 (1.0)	5 (5.0)
	**333**	**51**	**3**	**7**
**Colombia** (n=147)	66 (44.9)	73 (49.7)	5 (3.4)	3 (2.0)
**Panama** (n=186)	39 (21.0)	127 (68.3)	15 (8.1)	5 (2.7)
**Venezuela** (n=59)	26 (44.1)	26 (44.1)	3 (5.1)	4 (6.8)
**Puerto Rico** (n=202)	79 (39.1)	96 (47.5)	17 (8.4)	10 (5.0)
**Dominican Republic** (n=79)	14 (17.7)	50 (63.3)	12 (15.25)	3 (3.8)
	**224**	**372**	**52**	**25**
**Mexico** (n=65)	16 (24.6)	42 (64.6)	2 (3.1)	5 (7.7)
**Peru** (n=65)	12 (18.5)	47 (72.3)	-	6 (9.2)
**Ecuador** (n=15)	-	13 (86.7)	-	2 (13.3)
**Costa Rica** (n=96)	64 (66.7)	25 (26.0)	2 (2.1)	5 (5.2)
	**92**	**127**	**4**	**18**
**N= 1,308**	**649**	**550**	**59**	**50**
**%**	**49.6**	**42.1**	**4.5**	**3.8**

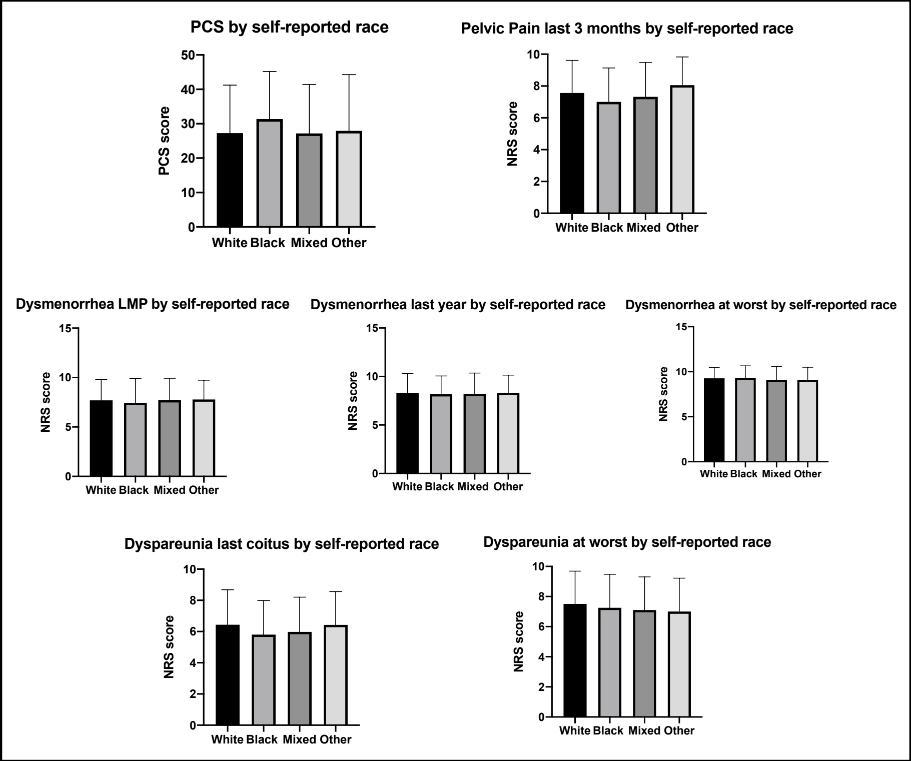

PCS by self-reported race
PCS score
50
40
30
20
10
0
White Black Mixed Other
Pelvic Pain last 3 months by self-reported race
NRS score
10
8
6
4
2
0
White Black Mixed Other
Dysmenorrhea LMP by self-reported race
NRS score
15
10
5
0
White Black Mixed Other
Dysmenorrhea last year by self-reported race
NRS score
15
10
5
0
White Black Mixed Other
Dysmenorrhea at worst by self-reported race
NRS score
15
10
5
0
White Black Mixed Other
Dyspareunia last coitus by self-reported race
NRS score
10
8
6
4
2
0
White Black Mixed Other
Dyspareunia at worst by self-reported race
NRS score
10
8
6
4
2
0
White Black Mixed Other

	Variables in the equation						
	**Age**	**Race (overall)**	**Race (White)**	**Race (Mixed)**	**Race (Black)**	**PCS Total Score**	**Constant**
Dysmenorrhea at last menstruation							
B	-0.004		-0.185	0.152	0.287	0.065	-1.193
SE	0.013		0.445	0.183	0.474	0.007	0.506
Wald	0.091	1.277	0.173	0.686	0.367	87.858	5.556
df	1	3	1	1	1	1	1
Sig	0.763	0.735	0.678	0.407	0.544	0.000	0.018
Dysmenorrhea last 12 months							
B	-0.022		-0.536	-0.038	-0.075	0.072	0.093
SE	0.014		0.459	0.202	0.515	0.008	0.554
Wald	2.395	1.373	1.363	0.036	0.021	82.562	0.028
df	1	3	1	1	1	1	1
Sig	0.122	0.712	0.243	0.849	0.885	0.000	0.866
Dysmenorrhea at worst							
B	-0.001		-0.590	-0.531	0.926	0.076	0.963
SE	0.016		0.573	0.244	1.051	0.010	0.644
Wald	0.004	6.416	1.060	4.742	0.775	54.643	2.238
df	1	3	1	1	1	1	1
Sig	0.947	0.093	0.303	0.029	0.379	0.000	0.135
Dyspareunia at last coitus							
B	0.015		-1.477	-0.384	-0.754	0.033	-2.033
SE	0.015		0.784	0.209	0.684	0.008	0.597
Wald	1.032	6.988	3.552	3.377	1.213	17.178	11.601
df	1	3	1	1	1	1	1
Sig	0.310	0.072	0.059	0.066	0.271	0.000	0.131
Dyspareunia 24 hours after coitus							
B	0.008		-1.195	-0.255	0.894	0.042	-2.289
SE	0.016		0.803	0.225	0.596	0.009	0.647
Wald	0.274	6.134	2.214	1.281	2.252	21.584	12.513
df	1	3	1	1	1	1	1
Sig	0.601	0.105	0.137	0.258	0.133	0.000	0.000
Dyspareunia at worst							
B	0.022		-0.667	-0.514	0.689	0.030	-1.155
SE	0.012		0.390	0.165	0.592	0.006	0.461
Wald	3.657	13.540	2.922	9.692	1.355	25.233	6.289
df	1	3	1	1	1	1	1
Sig	0.056	0.004	0.087	0.002	0.244	0.000	0.012
Pelvic pain last 3 months							
B	0.014		-0.652	-0.189	0.452	0.055	-1.863
SE	0.013		0.481	0.183	0.544	0.007	0.514
Wald	1.305	3.574	1.834	1.067	0.689	59.810	13.134
df	1	3	1	1	1	1	1
Sig	0.253	0.311	0.176	0.302	13.134	0.000	0.000
